# Vitamin D to prevent acute lung injury following oesophagectomy (VINDALOO): study protocol for a randomised placebo controlled trial

**DOI:** 10.1186/1745-6215-14-100

**Published:** 2013-04-17

**Authors:** Dhruv Parekh, Rachel C A Dancer, Sian Lax, Mark S Cooper, Adrian R Martineau, William D Fraser, Olga Tucker, Derek Alderson, Gavin D Perkins, Fang Gao-Smith, David R Thickett

**Affiliations:** 1 College of Medical and Dental Sciences, University of Birmingham, Vincent Drive, Birmingham B15 2TT, UK; 2 Warwick Medical School Clinical Trials Unit, University of Warwick, Gibbet Hill Road, Warwick, Coventry CV4 7AL, UK; 3 Centre for Primary Care and Public Health, Barts and The London School of Medicine and Dentistry, Queen Mary University of London, St Dunstan’s Road, London E1 2AB, UK; 4 Norwich Medical School, University of East Anglia, University Drive, Norwich NR4 7TJ, UK

**Keywords:** Acute lung injury, One lung ventilation, Oesophagectomy, Vitamin D

## Abstract

**Background:**

Acute lung injury occurs in approximately 25% to 30% of subjects undergoing oesophagectomy. Experimental studies suggest that treatment with vitamin D may prevent the development of acute lung injury by decreasing inflammatory cytokine release, enhancing lung epithelial repair and protecting alveolar capillary barrier function.

**Methods/Design:**

The ‘Vitamin D to prevent lung injury following oesophagectomy trial’ is a multi-centre, randomised, double-blind, placebo-controlled trial. The aim of the trial is to determine in patients undergoing elective transthoracic oesophagectomy, if pre-treatment with a single oral dose of vitamin D_3_ (300,000 IU (7.5 mg) cholecalciferol in oily solution administered seven days pre-operatively) compared to placebo affects biomarkers of early acute lung injury and other clinical outcomes. The primary outcome will be change in extravascular lung water index measured by PiCCO® transpulmonary thermodilution catheter at the end of the oesophagectomy. The trial secondary outcomes are clinical markers indicative of lung injury: PaO_2_:FiO_2_ ratio, oxygenation index; development of acute lung injury to day 28; duration of ventilation and organ failure; survival; safety and tolerability of vitamin D supplementation; plasma indices of endothelial and alveolar epithelial function/injury, plasma inflammatory response and plasma vitamin D status. The study aims to recruit 80 patients from three UK centres.

**Discussion:**

This study will ascertain whether vitamin D replacement alters biomarkers of lung damage following oesophagectomy.

**Trial registration:**

Current Controlled Trials ISRCTN27673620

## Background

Acute lung injury (ALI) and the more severe acute respiratory distress syndrome (ARDS) are common, devastating clinical syndromes of acute respiratory failure in the critically ill person. The incidence of ALI is 79 per 100,000 person years with a mortality rate of 30% to 65% [1]. Survivors of ARDS experience a significant reduction in health-related quality of life, with 46% reported to be unable to return to work within 12 months.

ALI is the final common pathway of response to a variety of direct pulmonary insults, such as bacterial /viral pneumonia and gastric aspiration, or indirect insults, such as abdominal sepsis or battlefield trauma. Only a relatively small proportion of patients develop ALI, with research suggesting that genetic, demographic (age), social (smoking, alcohol abuse) and other factors play a role in determining who develops ALI [1,2]. There are no current readily available tests that can clearly identify those who are at high risk of ALI and no therapeutic interventions proven to prevent its occurrence.

### One lung ventilation (OLV) as a model for ALI/ARDS

To allow access to the oesophagus during surgery (using the transthoracic technique), one of the lungs is deflated and the subject is ventilated through the other lung. This is known as one-lung ventilation (OLV). There is a high postoperative incidence of ALI/ARDS [3-5] following OLV and unlike most insults leading to lung injury the delivery of OLV is predictably timed, thereby allowing serial studies to be carried out throughout the period of stimulus and development of the condition. Preoperative risk factors including age, respiratory function and cigarette smoking have been found to be related to the incidence of postoperative pulmonary complications [3,6-8]*.* It is unclear at present why only a percentage of patients undergoing OLV develop lung injury or why the lung injury typically occurs 24 to 48 hours after the cessation of OLV. Our data show that the development of lung injury is, however, associated with a doubling of in-hospital stay and elevated mortality.

We have extensively modeled the local and systemic inflammatory response to transthoracic oesophagectomy in 50 patients undergoing OLV. After OLV, patients have a neutrophilic alveolitis, with a significant alveolar and systemic inflammatory response. This is associated with the release of markers of both endothelial and alveolar epithelial dysfunction and an increase in the permeability of the alveolar barrier. This manifests clinically as increased extravascular lung water and a fall in oxygenation.

Alveolar levels of surfactant protein D and bronchoalveolar lavage fluid (BALF) protein permeability index are highest in those who develop ALI within 72 hours of OLV suggesting that peri-operative alveolar epithelial damage is a risk factor for the subsequent development of ALI. Immediate post-operative plasma markers of neutrophilic activation (myeloperoxidase, and matrix metalloproteinase-9 (MMP-9)) as well as the receptor for advanced glycation end-products (RAGE, a type I epithelial cell marker) are similarly raised in those who develop ALI within 72 hours of OLV. Proposed causative mechanisms for this injury include the ischaemic/reperfusion insult suffered by the collapsed lung, as well as oxidative stress and barotrauma causing epithelial injury to the ventilated lung [9]. These mechanisms are important in the pathogenesis of ALI making OLV a valid model for studying the pathogenesis of ALI in humans and exploring therapeutic strategies for preventing lung injury in a predefined subject population [10,11].

### Vitamin D biology

Vitamin D_3_, or cholecalciferol, is mainly formed in the skin after exposure to sunlight, then hydroxylated in the liver to 25-hydroxyvitamin D_3_ (25(OH)D_3_) and subsequently in the kidney to 1,25-dihydroxyvitamin D_3_ (1,25(OH)_2_D_3_). When 25(OH)D_3_ is sufficiently available, 24,25-dihydroxyvitamin D_3_ (24,25(OH)_2_D_3_) is formed in the kidney which is further catabolised. Vitamin D metabolites are bound in the circulation to vitamin D binding protein (VDBP) which has a high affinity for 25(OH)D_3_, 24,25(OH)_2_D_3_ and 1,25(OH)_2_D_3_ and, therefore, regulates free circulating concentrations of vitamin D metabolites. The biologically active metabolite 1,25(OH)_2_D_3_ can also be generated locally within tissues due to induction of extra-renal cyp27b1 (25(OH)D-1-alpha hydroxylase) and binds to the vitamin D receptor (VDR) resulting in modified gene expression.

Epidemiological studies have suggested a role for low vitamin D status in the risk of developing both viral and bacterial infection [12,13]. A recent study has further demonstrated the pleiotropic anti-inflammatory effects of vitamin D in patients with pulmonary tuberculosis [14]. Published data suggest that vitamin D deficiency is common in critically ill patients [15], and recent prospective studies suggest an association with increased morbidity and mortality [16-18]. Literature on acute vitamin D supplementation in critical illness is lacking but serious adverse events attributable to vitamin D supplementation are rare [18,19].

### Is severe vitamin D deficiency a driver of post-OLV ALI?

Vitamin D has profound effects on human immunity acting as an immune system modulator, preventing excessive expression of inflammatory cytokines and increasing the ‘oxidative burst’ potential of macrophages, thereby enhancing bacterial killing. Vitamin D also stimulates the release of antimicrobial peptides such as LL-37 (cathelicidin) within the lung. LL-37 can also bind to and neutralize lipopolysaccharide (LPS), and functions as a chemoattractant for neutrophils, monocytes and T cells through a formyl peptide receptor-like molecule [20].

Respiratory epithelial cells convert 25(OH)D_3_ to 1,25(OH)_2_D_3_ and activate VDR responsive genes increasing the production of *hCAP18* from which LL-37 is cleaved within 24 hours [21]. In terms of the pathophysiology of ALI this could be important as LL-37 may drive epithelial repair responses as well as being an anti-microbial peptide [22]. Elevating local LL-37 may also be important as a downstream immunomodulator of vitamin D since it has recently been shown to reduce Toll-like receptor (TLR) agonist-mediated neutrophil-derived increases in IL-1β, IL-6, IL-8 and tumour necrosis factor-alpha (TNF-α) in addition to stimulating bacterial phagocytosis [23]. The ability of 1,25(OH)_2_D_3_ to directly inhibit nuclear factor-kappaB (NF-kb) signalling and suppress macrophage TLR expression suggests that vitamin D may also play a key role as a feedback regulator of macrophage responses [24,25].

The only study looking at vitamin D levels in patients with severe sepsis suggests that these patients have a lower serum vitamin D level than healthy control patients. This was associated with lower plasma levels of LL-37, suggesting that this deficiency is of functional importance *in vivo*[26]. IL-1 and TNF production induced by TLR3 agonists from monocyte derived macrophages are inhibited to the same extent by 25(OH)D_3_ and 1,25(OH)_2_D_3_ after 24 hours suggesting that the vitamin D metabolites may have a rapid anti-inflammatory action and that local intracrine activation of 25(OH)D_3_ can be anti-inflammatory [27].

Recent data have further implicated vitamin D in adaptive immunity because of its influence upon the differentiation of T cells between the regulatory T cell (T_reg_) and the pro-inflammatory T helper 17 (T_h_17) subsets [28-30]. T_h_17 cells are known to stimulate tissue inflammation and neutrophil chemotaxis, both of which are seen in ALI, predominantly by IL-17 production. It also appears that expression of markers of T_reg_ cells (Foxp3) or T_h_17 cells (IL-17) by T cells may not be stable and that there is a greater degree of plasticity in their differentiation than previously appreciated [29]. Recent evidence has further suggested T_reg_ cells are important in the resolution of experimental ALI. This suggests that local lung regulation of the balance between T_reg_ and T_h_17 cells may be a determinant of resolution/persistence of neutrophilic inflammation which is known to be associated with a poor prognosis in human ALI.

Although the above suggest a potentially beneficial effect of vitamin D, we must exercise some caution as some studies have shown potentially adverse effects of vitamin D. In low dose nasal LPS challenge, cellular inflammation is actually lower in vitamin D receptor deficient (VDR KO) mice due to toll-receptor hyporesponsiveness. In addition the chemotactic effects of LL-37 could in theory increase neutrophil recruitment to the lung; albeit in ALI, CXCL-8 and ENA-78 are the main chemokines driving neutrophil recruitment. More recently it has been shown that the plasma LL-37 concentration was decreased in vitamin D supplemented patients with tuberculosis, possibly representing a global suppressive effect of vitamin D supplementation on markers of acute phase response or an indirect response to enhanced microbial killing [14]. Despite these reservations, the predominant biological effects of vitamin D led us to hypothesise that vitamin D deficiency may be a risk factor for ALI, causing elevated inflammation which results in exaggerated epithelial damage in at risk, vitamin D deficient individuals.

The VINDALOO trial is a three centre randomised double blind, placebo-controlled trial aiming to define the safety and effectiveness of a single high dose of vitamin D in preventing ALI in a group of patients at high risk of developing the condition.

## Methods/Design

### Trial approvals and conduct

The trial is approved by South Birmingham Research Ethics Committee (REC 12/WM/0092). The trial is registered on the International Standard Randomised Controlled Trial Registry (ISRCTN27673620). The sponsor organisation for the trial is the University of Birmingham. The trial is funded by the Medical Research Council UK (MRC reference G1100196). The trial will be carried out in accordance with the Medical Research Council (MRC) Good Clinical Practice Guidelines, applicable UK legislation and Standard Operating Procedures of the Perioperative and Critical Care Trials Group at the University of Birmingham. The trial will be reported in line with the Consolidated Standards of Reporting Trials (CONSORT) 2010 guidelines [31].

### Outcome measures

#### Primary outcome

The primary outcome will be the extravascular lung water index (EVLWI) measured by PiCCO thermodilution catheter at the end of the oesophagectomy (measured within one hour post-operatively). In ALI the changes in lung compliance that are a cardinal feature of this disease occur due to the accumulation of extravascular lung water (EVLW). The PiCCO EVLWI has been shown to be an independent risk factor for mortality in ALI and has been used as the primary outcome in several clinical trials in ALI (BALTI-1, HARP) [32,33] as well as post-thoracotomy [34]. In BALTI-1 we demonstrated that the transpulmonary thermodilution technique (PiCCO) is able to detect a significant change in lung water with a treatment that might be expected to achieve this objective. We have tested the CV coefficient of variance of EVLWI measurements previously and found it to be 6.8% over six sequential assessments over two hours. Further, we have studied the perioperative changes in lung water following oesophagectomy and demonstrated that preoperative vitamin D status influences the level of accumulation of EVLW.

### Secondary outcomes

The trial secondary outcomes are clinical markers indicative of lung injury: PaO_2_:FiO_2_ ratio, oxygenation index, development of lung injury/ARDS during the first 28 days, ventilator and organ failure free days, survival (28 and 90 day) and safety and tolerability of vitamin D supplementation.

Lung injury will be defined by the American European Consensus Conference definition [35] as the acute onset of: (1) bilateral infiltrates on the chest x-ray; (2) hypoxaemia with a PaO_2_:FiO_2_ ratio of <40 kPa; and (3) absence of clinical evidence of left atrial hypertension. Ventilator free days are defined in accordance with the ARDSnet criteria as the number of calendar days after initiating unassisted breathing to day 28 after randomisation, assuming a patient survives for at least 48 consecutive hours after initiating unassisted breathing [36]. Un-assisted breathing is defined as one of at least 48 consecutive hours of: (1) being extubated with face mask, nasal prong oxygen, or room air; (2) T-tube breathing; and (3) tracheostomy mask breathing, CPAP = 5 cm H_2_0 without pressure support of intermittent mandatory ventilation assistance.

Organ failure free days are defined in a similar manner to ventilator free days with an organ failure free day being a day without evidence of non-respiratory organ failure. Organ failure will be defined as a sequential organ failure assessment (SOFA) score of greater than three [37].

Plasma indices of endothelial and alveolar epithelial function/injury, plasma inflammatory response will be measured by ELISA. Plasma vitamin D status (25(OH)D_3_, 1,25(OH)_2_D_3_, VDBP) and calcium will be assessed peri-operatively (pre-drug dose, pre-operative, post-operative, day 1 and day 3) as well as EVLWI day 1 post-operatively (measured at 9 am on day 1).

### Eligibility criteria

Patients will be eligible for the trial if they fulfill the following criteria:

• Planned transthoracic oesophagectomy for oesophageal carcinoma at a participating centre.

• Men over 18 years old on the day of first dose of the investigational medicinal product (IMP).

• Women over the age of 55 or more than 2 years since menopause.

• Women of potential child bearing age (under 55 and less than two years since menopause) may be recruited provided they agree to use contraception during the pre-post-operative period (eight weeks).

• Ability to give written informed consent to participate in the study.

Patients fulfilling any of the criteria below will be excluded:

• Known intolerance of vitamin D.

• Known sarcoidosis, hyperparathyroidism, or nephrolithiasis.

• Known serum corrected calcium >2.65 mmol/L.

• Undergoing haemodialysis.

• Pregnant or breastfeeding.

• Patients with tuberculosis or lymphoma.

• Diagnosis of chronic obstructive pulmonary disease (COPD) with a forced expiratory volume in one second (FEV1) less than 50% predicted or resting oxygen saturations of less 92%.

### Power and sample size estimate

There are no direct data to predict the effect size of vitamin D replacement upon EVLWI. In our preliminary work with 50 patients, the patients with 25(OH)D_3_ concentrations less than 15 nmol/L had the greatest increases in EVLWI (+3.2 ml/kg, +27%) compared to those patients with less severe deficiency pre–postoperatively (+1.0 ml/kg, +10% *P* = 0.013) suggesting that severe vitamin D deficiency influences EVLWI. As a group, after undergoing oesophagectomy our patients have a mean post-operative EVLWI of 10.1 ml/kg and standard deviation of 2.9 ml/kg. For the study to be able to detect a change of 20% in EVLWI with a power of 80% we will require approximately 34 patients in each arm to reach the primary endpoint (*P* = 0.05). An additional six patients will be needed to allow for dropouts, such as open/close cases, unexpected deaths and other difficulties with data collection. Thus, we intend to recruit 40 patients to each arm of the study.

With 34 patients completing each arm of the study, based upon preliminary unpublished data from 50 oesophagectomy cases, this study would detect a treatment effect of vitamin D upon the PO_2_:FiO_2_ ratio postoperative (PO) of ± 8.16 (± 20%), PO plasma soluble intercellular adhesion molecule-1 (sICAM-1) of ± 16 ng/ml (± 32%), plasma C-reactive protein (CRP) ± 53 ng/ml (± 33%), PO plasma von Willebrand factor (vWF) ± 55% relative to control plasma (± 24.7% change), PO plasma high-mobility group box 1 (HMGB-1) 5.42 ng/ml (± 54%), PO plasma myeloperoxidase (MPO) 105 pg/ml (± 57%), PO plasma MMP-9 51 ng/ml (± 57%), and PO plasma surfactant protein-D (SP-D) 691 ng/ml (± 61%). All calculations assume 80% power at a two-tailed significance level of 0.05.

### Trial conduct

#### Approach to patients and obtaining informed consent

Patients will be identified through upper gastrointestinal cancer teams. Eligible patients will be invited to participate by their treating clinician, specialist clinical nurse or research nurse. If agreeable, written informed consent will be obtained, following a face to face discussion about the study.

### Randomisation and drug / placebo supply

The trial drug manufacturer will produce the randomisation sequence using a block size of 10 with equal allocation between active and placebo groups. Drug (oral cholecalciferol oily solution Vigantol, 300,000 IU (7.5 mg)) and matching placebo (Miglyol 812 oil, the vehicle for vitamin D3 in Vigantol) will be supplied and packaged according to the randomisation sequence into numbered treatment boxes by Novalabs (Leicester, UK). Drug boxes will be supplied to centres in blocks of 10 thus ensuring an equal allocation between active and placebo groups to balance any differences in case mix, pre-operative, operative and post-operative care between centres. Patients will be randomised sequentially by allocating them to the next numbered treatment pack held at the centre.

### Drug administration

Subjects will receive either 300,000 IU (7.5 mg) of vitamin D or placebo seven days prior to planned oesophagectomy. The drug will be administered by qualified medical or nursing staff.

### Concomitant medications

Patients taking the following medications are not eligible for inclusion in the study:

• Taking more than 1,000 IU/day (25 mcg/day) vitamin D supplementation by month preceding enrolment.

• Taking cardiac glycoside, carbamazepine, phenobarbital, phenytoin, primidone or long-term immunosuppressant therapy.

• Patients taking benzothiadiazine derivatives at doses higher than that which is recommended in the British National Formulary (BNF).

• Patients taking a benzothiadiazine derivative in combination with a calcium supplement.

### Post randomisation withdrawals and exclusions

Subjects may withdraw from the trial or the trial treatment at any time without prejudice. If a subject withdraws from the trial treatment, then they will be followed-up wherever possible and data collected as per protocol until the end of the trial. The only exception to this is where the subject also explicitly withdraws consent for follow-up.

### Blinding/un-blinding

Patients, clinical and research / trial staff will be unaware of the arm of the study to which a patient is allocated. Active and placebo treatment packs and their contents will be identical in appearance. The protocol allows for emergency un-blinding in the event of significant concerns about patient safety. In the unlikely event that un-blinding is required the local investigator will discuss this with the Chief Investigator. All events will be logged.

### Monitoring and reporting adverse events

VINDALOO is recruiting a population who are prone to recognized medical and surgical complications. It is expected that many of the patients will experience an event that might be seen as a serious adverse event but is a recognized complication following oesophagectomy. Adverse and serious adverse events which are recognised complications of surgery, for example, medical (pneumonia, sepsis) and surgical (chyle leak, anastamosis leak) will be recorded in the case report form.

Death and other serious adverse events thought to be related to the study drug or serious unexpected serious adverse reactions will be reported to the trial co-ordinating centre and Chief Investigator within 24 hours of becoming aware of their occurrence. The Chief Investigator will inform the sponsor and regulatory authorities.

### Data collection

Data up until hospital discharge will be recorded in each subject's Case Report Form (CRF) by a member of the trials team. Most of the data collected will be obtained from the patient’s hospital notes. In the unlikely event that a subject is transferred to another hospital, the study team will ensure that data collection is completed by the receiving hospital.

If the subject remains in hospital at 28 or 90 days, survival at these time points will be recorded by hospital staff. Mortality after hospital discharge will be obtained from the National Health Service (NHS) Statistical Tracing Service (NSTS).

### Statistical analysis plan

Data will be analysed with the help of the trial statistician. Data will be analysed using SPSS for Windows 17.0. A detailed analysis plan will be developed during the trial prior to commencement of analysis. In brief, for continuously distributed data, differences between groups will be tested using independent samples t-tests with transformations of variables to Normality if appropriate, or non-parametric equivalents. Chi-squared tests (or Fisher’s Exact tests) will be used for categorical variables. A *P* value of 0.05 will be considered as significant. We will test for significant correlations between changes in the biological markers using standard methods. The treatment effect will be analysed on an intention-to-treat basis. For further examinations of relationships between a binary variable and known explanatory variables, the following tests will be applied as appropriate. Logistic regression will be used to provide the estimated risk ratios for the treatment effect with associated 95% confidence interval (CI). Time to event outcomes, such as duration of ventilation or duration of hospital stay, will be analysed by survival methods and reported as hazard ratios and 95% CI. A single final analysis is planned at the end of the trial.

### Trial organisation/oversight

Trial oversight will be provided by a Trial Steering Committee (TSC) comprising investigators, clinicians and trialists. An independent data monitoring committee will monitor the safety of participants enrolled in the trial through regular review of adverse event reports. An interim analysis of efficacy is not planned.

## Discussion

The preliminary data that this study is based upon suggests a significant role for vitamin D deficiency as a risk factor for the development of acute lung injury. This trial will look at biomarkers of alveolar epithelial damage and inflammation to determine the proof of concept that vitamin d replacement may have efficacy as a preventative agent for the development of acute lung injury.

## Trial status

Patient recruitment commenced in September 2012 and is expected to run for two years (last patient recruited in August 2014).

## Abbreviations

ALI: Acute lung injury; ARDS: Acute respiratory distress syndrome; BAL: Bronchoalveolar lavage; CI: Confidence interval; DBP: Vitamin D binding protein; ELISA: Enyme-linked immunosorbent assay; EVLWI: Extravascular lung water index; IL: Interleukin; IMP: Investigational Medicinal Product; ISRCTN: International Standard Randomised; MMP-9: Matrix metalloproteinase-9; MRC: Medical Research Council; OLV: One lung ventilation; PICCO: Pulse Contour Cardiac Output Monitoring; RAGE: Receptor for advanced glycation endpoints; REC: Research Ethics Committee; TNF-α: Tumour necrosis factor alpha; Th: T helper cells; Treg: Regulatory T cells; VDBP: Vitamin D binding protein; VDR: Vitamin D receptor; 25(OH)D: 25-hydroxyvitamin D.

## Competing interests

The authors declare that they have no competing interests.

## Authors’ contributions

All authors made a substantial contribution to the protocol development. All authors have read and approved this manuscript.

## References

[B1] Lewandowski K Lewandowski M Epidemiology of ARDS Minerva Anestesiol 2006 72 473 477 16682918

[B2] Rubenfeld GD Caldwell E Peabody E Weaver J Martin DP Neff M Stern EJ Hudson LD Incidence and outcomes of acute lung injury N Engl J Med 2005 353 1685 1693 10.1056/NEJMoa050333 16236739

[B3] Tandon S Batchelor A Bullock R Gascoigne A Griffin M Hayes N Hing J Shaw I Warnell I Baudouin S Peri-operative risk factors for acute lung injury after elective oesophagectomy Br J Anaesth 2001 86 633 638 10.1093/bja/86.5.633 11575337

[B4] Schilling M Gassmann N Sigurdsson G Regli B Stoupis C Furrer M Signer C Redaelli C Büchler M Role of thromboxane and leukotriene B4 in patients with acute respiratory distress syndrome after oesophagectomy Br J Anaesth 1998 80 36 40 10.1093/bja/80.1.36 9505775

[B5] Schilling M Eichenberger M Maurer C Sigurdsson G Büchler M Ketoconazole and pulmonary failure after esophagectomy: a prospective clinical trial Dis Esophagus 2001 14 37 40 10.1111/j.1442-2050.2001.00158.x 11422304

[B6] Kuwano H Sumiyoshi K Sonoda K Kitamura K Tsutsui S Toh Y Kitamura M Sugimachi K Relationship between preoperative assessment of organ function and postoperative morbidity in patients with oesophageal cancer Eur J Surg 1998 164 581 586 9720934 10.1080/110241598750005679

[B7] Ferguson M Durkin A Preoperative prediction of the risk of pulmonary complications after esophagectomy for cancer J Thorac Cardiovasc Surg 2002 123 661 669 10.1067/mtc.2002.120350 11986593

[B8] Law S Wong K Kwok K Chu K Wong J Predictive factors for postoperative pulmonary complications and mortality after esophagectomy for cancer Ann Surg 2004 240 791 800 10.1097/01.sla.0000143123.24556.1c 15492560 PMC1356484

[B9] Baudouin S Lung injury after thoracotomy Br J Anaesth 2003 91 132 142 10.1093/bja/aeg083 12821572

[B10] Ware LB Matthay MA The acute respiratory distress syndrome N Engl J Med 2000 342 1334 1349 10.1056/NEJM200005043421806 10793167

[B11] Perkins GD Park D Alderson D Cooke MW Gao F Gates S Lamb SE Mistry D Thickett DR The Beta Agonist Lung Injury TrIal (BALTI) - prevention trial protocol Trials 2011 12 79 10.1186/1745-6215-12-79 21406094 PMC3068101

[B12] Cannell JJ Vieth R Umhau JC Holick MF Grant WB Madronich S Garland CF Giovannucci E Epidemic influenza and vitamin D Epidemiol Infect 2006 134 1129 1140 10.1017/S0950268806007175 16959053 PMC2870528

[B13] Muhe L Lulseged S Mason KE Simoes EA Case–control study of the role of nutritional rickets in the risk of developing pneumonia in Ethiopian children Lancet 1997 349 1801 1804 10.1016/S0140-6736(96)12098-5 9269215

[B14] Coussens AK Wilkinson RJ Hanifa Y Nikolayevskyy V Elkington PT Islam K Timms PM Venton TR Bothamley GH Packe GE Darmalingam M Davidson RN Milburn HJ Baker LV Barker RD Mein CA Bhaw-Rosun L Nuamah R Young DB Drobniewski FA Griffiths CJ Martineau AR Vitamin D accelerates resolution of inflammatory responses during tuberculosis treatment Proc Natl Acad Sci U S A 2012 109 15449 15454 10.1073/pnas.1200072109 22949664 PMC3458393

[B15] Lee P Eisman JA Center JR Vitamin D deficiency in critically ill patients N Engl J Med 2009 360 1912 1914 10.1056/NEJMc0809996 19403914

[B16] Braun A Chang D Mahadevappa K Gibbons FK Liu Y Giovannucci E Christopher KB Association of low serum 25-hydroxyvitamin D levels and mortality in the critically ill Crit Care Med 2011 39 671 677 10.1097/CCM.0b013e318206ccdf 21242800 PMC3448785

[B17] Braun AB Gibbons FK Litonjua AA Giovannucci E Christopher KB Low serum 25-hydroxyvitamin D at critical care initiation is associated with increased mortality Crit Care Med 2012 40 63 72 10.1097/CCM.0b013e31822d74f3 21926604 PMC4427350

[B18] Amrein K Sourij H Wagner G Holl A Pieber TR Smolle KH Stojakovic T Schnedl C Dobnig H Short-term effects of high-dose oral vitamin D3 in critically ill vitamin D deficient patients: a randomized, double-blind, placebo-controlled pilot study Crit Care 2011 15 R104 10.1186/cc10120 21443793 PMC3219377

[B19] Hughes DA Norton R Vitamin D and respiratory health Clin Exp Immunol 2009 158 20 25 10.1111/j.1365-2249.2009.04001.x 19737226 PMC2759054

[B20] Zanetti M Cathelicidins, multifunctional peptides of the innate immunity J Leukoc Biol 2004 75 39 48 12960280 10.1189/jlb.0403147

[B21] Hansdottir S Monick MM Lovan N Powers L Gerke A Hunninghake GW Vitamin D decreases respiratory syncytial virus induction of NF-kappaB-linked chemokines and cytokines in airway epithelium while maintaining the antiviral state J Immunol 2010 184 965 974 10.4049/jimmunol.0902840 20008294 PMC3035054

[B22] Shaykhiev R Beisswenger C Kandler K Senske J Puchner A Damm T Behr J Bals R Human endogenous antibiotic LL-37 stimulates airway epithelial cell proliferation and wound closure Am J Physiol Lung Cell Mol Physiol 2005 289 L842 L848 10.1152/ajplung.00286.2004 15964896

[B23] Alalwani SM Sierigk J Herr C Pinkenburg O Gallo R Vogelmeier C Bals R The antimicrobial peptide LL-37 modulates the inflammatory and host defense response of human neutrophils Eur J Immunol 2010 40 1118 1126 10.1002/eji.200939275 20140902 PMC2908514

[B24] Sun J Kong J Duan Y Szeto FL Liao A Madara JL Li YC Increased NF-kappaB activity in fibroblasts lacking the vitamin D receptor Am J Physiol Endocrinol Metab 2006 291 E315 E322 10.1152/ajpendo.00590.2005 16507601

[B25] Sadeghi K Wessner B Laggner U Ploder M Tamandl D Friedl J Zugel U Steinmeyer A Pollak A Roth E Boltz-Nitulescu G Spittler A Vitamin D3 down-regulates monocyte TLR expression and triggers hyporesponsiveness to pathogen-associated molecular patterns Eur J Immunol 2006 36 361 370 10.1002/eji.200425995 16402404

[B26] Jeng L Yamshchikov AV Judd SE Blumberg HM Martin GS Ziegler TR Tangpricha V Alterations in vitamin D status and anti-microbial peptide levels in patients in the intensive care unit with sepsis J Transl Med 2009 7 28 10.1186/1479-5876-7-28 19389235 PMC2684740

[B27] Herr C Branschied M Vogelmeier C Bals R Influence of Vitamin D on the innate immune response in cigarette smoke exposed macrophages Eur Respir Soc Barcelona meet 2010 A4349

[B28] Zhou X Bailey-Bucktrout S Jeker LT Bluestone JA Plasticity of CD4(+) FoxP3(+) T cells Curr Opin Immunol 2009 21 281 285 10.1016/j.coi.2009.05.007 19500966 PMC2733784

[B29] Zhou L Chong MM Littman DR Plasticity of CD4+ T cell lineage differentiation Immunity 2009 30 646 655 10.1016/j.immuni.2009.05.001 19464987

[B30] Jeffery LE Burke F Mura M Zheng Y Qureshi OS Hewison M Walker LS Lammas DA Raza K Sansom DM 1,25-Dihydroxyvitamin D(3) and IL-2 combine to inhibit T cell production of inflammatory cytokines and promote development of regulatory T cells expressing CTLA-4 and FoxP3 J Immunol 2009 183 5458 5467 10.4049/jimmunol.0803217 19843932 PMC2810518

[B31] Schulz KF Altman DG Moher D CONSORT 2010 statement: updated guidelines for reporting parallel group randomised trials J Pharmacol Pharmacother 2010 1 100 107 10.4103/0976-500X.72352 21350618 PMC3043330

[B32] Perkins GD McAuley DF Thickett DR Gao F The beta-agonist lung injury trial (BALTI): a randomized placebo-controlled clinical trial Am J Respir Crit Care Med 2006 173 281 287 10.1164/rccm.200508-1302OC 16254268

[B33] Craig TR Duffy MJ Shyamsundar M McDowell C O'Kane CM Elborn JS McAuley DF A randomized clinical trial of hydroxymethylglutaryl- coenzyme a reductase inhibition for acute lung injury (The HARP Study) Am J Respir Crit Care Med 2011 183 620 626 10.1164/rccm.201003-0423OC 20870757

[B34] Licker M Tschopp JM Robert J Frey JG Diaper J Ellenberger C Aerosolized salbutamol accelerates the resolution of pulmonary edema after lung resection Chest 2008 133 845 852 10.1378/chest.07-1710 17989152

[B35] Artigas A Bernard GR Carlet J Dreyfuss D Gattinoni L Hudson L Lamy M Marini JJ Matthay MA Pinsky MR Spragg R Suter PM The American-European Consensus Conference on ARDS, part 2. Ventilatory, pharmacologic, supportive therapy, study design strategies and issues related to recovery and remodeling Intensive Care Med 1998 24 378 398 10.1007/s001340050585 9609420

[B36] Artigas A Bernard GR Carlet J Dreyfuss D Gattinoni L Hudson L Lamy M Marini JJ Matthay MA Pinsky MR Spragg R Suter PM The American-European Consensus Conference on ARDS, part 2: Ventilatory, pharmacologic, supportive therapy, study design strategies, and issues related to recovery and remodeling. Acute respiratory distress syndrome Am J Respir Crit Care Med 1998 157 1332 1347 10.1164/ajrccm.157.4.ats2-98 9563759

[B37] Ferreira FL Bota DP Bross A Melot C Vincent JL Serial evaluation of the SOFA score to predict outcome in critically ill patients JAMA 2001 286 1754 1758 10.1001/jama.286.14.1754 11594901

